# Construction and evaluation of a diagnostic prediction model for bacterial meningitis based on clinical and laboratory data

**DOI:** 10.3389/fneur.2026.1747753

**Published:** 2026-02-13

**Authors:** Xiaotong Shen, Lidan Xing, Shichao Gao, Yaomeng Huang, Xinhui Yu, Zheng Zhang

**Affiliations:** 1 Hebei Medical University, Shijiazhuang, China; 2 Clinical Laboratory Center, The First Hospital of Hebei Medical University, Shijiazhuang, China

**Keywords:** bacterial meningitis, diagnostic prediction model, etiological distribution, logistic regression, nomogram, ROC curve

## Abstract

Bacterial meningitis refers to the rapid inflammation of the meninges caused by bacteria or their byproducts, impacting the pia mater, arachnoid mater, and the subarachnoid space. This condition is a serious infectious illness affecting the central nervous system, if not diagnosed and treated promptly, it may result in severe neurological complications or even fatalities, making prompt and precise diagnosis essential for better outcomes. The objective of this research was to develop and assess a diagnostic prediction model for bacterial meningitis utilizing clinical and laboratory information. A retrospective study was carried out on patients with central nervous system infections who were admitted to the First Hospital of Hebei Medical University between January 2022 and February 2025. Both univariate and multivariate logistic regression analyses were utilized to create the prediction model, identifying key independent factors such as intracerebral hemorrhage, hydrocephalus, C-reactive protein (CRP), lymphocyte percentage (LY), cerebrospinal fluid chloride level (CSFCL), and the white blood cell count in cerebrospinal fluid. The results of logistic regression analysis were used to construct a nomogram to visualize the risk of bacterial meningitis in patients. The effectiveness of the model was assessed through calibration curves, the area under the receiver operating characteristic curve (AUC), and decision curve analysis (DCA). Findings indicated that the AUC for the prediction model was 0.84 (95% CI: 0.78–0.89) for the training cohort and 0.77 (95% CI: 0.66–0.87) for the validation cohort. In summary, this model exhibits strong diagnostic capabilities and serves as a valuable tool for the swift clinical identification of bacterial meningitis.

## 1 Introduction

Bacterial meningitis is a serious inflammatory condition affecting the central nervous system, triggered by bacterial infections (1–3). It typically presents suddenly and can escalate quickly; if not diagnosed and treated promptly, it may result in severe neurological complications or even fatalities (4). Annually, this illness accounts for over 300,000 deaths globally (5). Survivors often face considerable disabilities due to lasting neurological impairments (6, 7). Thus, timely and precise diagnosis is crucial for enhancing patient outcomes (8–10). Nevertheless, the aetiology of meningitis and encephalitis varies from infectious agents, including viruses, bacteria, mycobacteria, fungi, and parasites, to the autoimmune process, rendering early aetiologic diagnosis and rapid treatment a challenge, ultimately leading to a poor prognosis (11, 12). Aetiologic classification is confirmed based on culture and antibody test results and requires time and specific instrumentation and facilities. Thus, researchers attempted classification by studying the differences in the initial clinical presentation, brain imaging features, electroencephalography (EEG), and cerebrospinal fluid (CSF) findings among cases with different aetiologies (3, 13–16). Currently, diagnosing bacterial meningitis involves assessing clinical symptoms, conducting laboratory analyses, and performing imaging studies. Patients may not always exhibit the classic symptoms, such as neck stiffness, fever, and altered mental status, making cerebrospinal fluid (CSF) analysis vital for biochemical, microscopic, culture, and PCR testing to detect bacterial DNA (17). CSF molecular tests, on the other hand, have high specificity but consistently low sensitivity (18). Cerebrospinal fluid bacterial culture is the gold standard for diagnosis, but the widespread use of broad-spectrum antibiotics in the early stages of hospitalization often results in low positivity rates for cerebrospinal fluid pathogen cultures (19). Furthermore, differentiating between bacterial and viral meningitis can be challenging, often resulting in misdiagnoses.

This study employed a retrospective analysis approach to collect baseline data, clinical manifestations, and laboratory test results from patients with central nervous system infections at the First Hospital of Hebei Medical University. A predictive model was constructed using multivariate logistic regression analysis. This study aimed to construct a diagnostic model integrating clinical and laboratory data to differentiate between purulent and non-Bacterial meningitis patients, thereby improving diagnostic accuracy and timeliness.

## 2 Materials and methods

### 2.1 Patient data

This research utilized clinical and laboratory records from individuals diagnosed with bacterial meningitis, as well as control subjects, at the First Hospital of Hebei Medical University. The data collection spanned from January 2022 to February 2025, encompassing 264 patients with central nervous system infections, which included 137 instances of bacterial meningitis and 127 instances of non-bacterial meningitis. Meet the WHO criteria for confirmed bacterial meningitis: (1) Acute fever (axillary temperature >38.0 °C or rectal temperature >38.5 °C);(2) At the same time, one or more of the following clinical manifestations: headache; positive meningeal irritation signs; altered consciousness;(3)The presence of abnormalities in routine CSF examinations and CSF chemistry tests (such as elevated white blood cells and proteins) suggests the presence of intracranial infection.(4) Positive CSF or blood culture, positive CSF or blood pathogen antigen test, or positive Gram stain; cases with a clear pathogen, and excluding non-bacterial meningitis and tuberculous meningitis (20). Diagnostic Criteria for Non-Bacterial meningitis: patients with negative cerebrospinal fluid bacterial cultures, blood cultures, and no clear pathogen detected by metagenomic next-generation sequencing.

### 2.2 Data collection

Data on demographics such as age and sex, along with clinical manifestations like fever, headaches, nausea or vomiting, changes in consciousness, signs of meningeal irritation, intracerebral hemorrhage, hydrocephalus, diabetes, and hypertension, were gathered. Laboratory metrics comprised white blood cell count (WBC), percentages of neutrophils (NE%) and lymphocytes (LY%), levels of interleukin-6 (IL-6), rapid C-reactive protein (CRP), procalcitonin (PCT), as well as biochemical and routine tests of cerebrospinal fluid (CSF), including total cell count and white blood cell count (7).

### 2.3 Model construction

In the multivariate logistic regression, variables showing a significance level of *p* < 0.2 from the univariate analysis were selected using a backward stepwise approach. Both univariate and multivariate logistic regression analyses were conducted to develop the predictive model, pinpointing independent factors including intracerebral hemorrhage, hydrocephalus, CRP, LY, CSFCL, and the white blood cell count in cerebrospinal fluid.

### 2.4 Statistical analysis

Statistical analyses were conducted using SPSS 27.0 software. Categorical data were presented as counts and percentages, and comparisons between groups were performed using the chi-square test. Continuous data were compared using the t-test. The study population was randomly divided into training and validation sets in a 7:3 ratio. Univariate logistic regression analysis was performed, and variables with a *p*-value < 0.2 were included in the multivariate logistic regression analysis. Receiver operating characteristic (ROC) curves were constructed for the training and validation sets, and the predictive performance of the model was evaluated by calculating the area under the curve (AUC). The Hosmer-Lemeshow (H-L) test evaluated the model’s goodness-of-fit for both sets, while calibration curves were utilized to compare predicted outcomes with actual results. A nomogram was developed using R Studio, and decision curve analysis (DCA) was conducted to evaluate the model’s clinical utility.

## 3 Results

### 3.1 Demographic and clinical characteristics

Among the 137 patients with Bacterial meningitis, the male-to-female ratio was 1.63:1, with 85 males and 52 females. The peak age of onset was 50–64 years with 8 cases aged 0–12, 11 cases aged 13–19, 9 cases aged 20–34, 31 cases aged 35–49, 42 cases aged 50–64, and 36 cases aged 65 and older. The clinical symptoms and signs of the 137 Bacterial meningitis cases included fever (60 cases, 43.8%), headache (68 cases, 49.64%), nausea/vomiting (64 cases, 46.72%), altered consciousness (107 cases, 78.10%), meningeal irritation signs (61 cases, 44.53%), intracerebral hemorrhage (61 cases, 44.53%), hydrocephalus (28 cases, 20.44%), diabetes (36 cases, 26.28%), and hypertension (86 cases, 62.77%). The demographic and clinical profiles of the two groups are contrasted in Table 1; patients with bacterial meningitis were more likely to present with altered consciousness, intracerebral haemorrhage and hydrocephalus, indicating that the prediction model will be most useful in adults with acute central-nervous-system infection who show neuro-imaging abnormalities or impaired consciousness.

**Table 1:** Baseline demographic and clinical characteristics of the study population.

Characteristic	NPM (*n* = 127)	PM (*n* = 137)	*p*
Age, median (IQR)	49 (38–61)	52 (42–63)	0.18
Male sex, *n* (%)	71 (55.9)	85 (62.0)	0.29
Fever > 38 °C, *n* (%)	45 (35.4)	60 (43.8)	0.14
Headache, *n* (%)	58 (45.7)	68 (49.6)	0.52
Nausea/vomiting, *n* (%)	54 (42.5)	64 (46.7)	0.47
Altered consciousness, *n* (%)	73 (57.5)	107 (78.1)	**<0.001**
Meningeal irritation, *n* (%)	48 (37.8)	61 (44.5)	0.24
Intracerebral haemorrhage, *n* (%)	14 (11.0)	61 (44.5)	**<0.0001**
Hydrocephalus, *n* (%)	8 (6.3)	28 (20.4)	**0.001**
Diabetes, *n* (%)	29 (22.8)	36 (26.3)	0.49
Hypertension, *n* (%)	68 (53.5)	86 (62.8)	0.1

*p* values from χ^2^ test or Mann–Whitney U test. Significant differences (*p* < 0.05) are shown in bold.

### 3.2 Univariate analysis

Univariate analysis was performed to identify significant differences between Bacterial meningitis patients and non-patients. For continuous variables (e.g., fever, PCT, WBC), the Mann–Whitney U test was used to compare median differences between groups. For categorical variables (e.g., headache, nausea/vomiting), the chi-square test was used to compare distributions between groups. Variables with a *p*-value < 0.05 were considered statistically significant. The results showed significant differences in WBC, NE, LY, CRP, CSFP, CSFCL, cerebrospinal fluid cell count, fever, altered consciousness, meningeal irritation signs, hydrocephalus, intracerebral hemorrhage, and hypertension between Bacterial meningitis patients and non-patients (Table 2).

**Table 2:** Distribution differences of indicators between bacterial and nonbacterial meningitis.

Variables	NPM (*n* = 127)	PM (*n* = 137)	Statistic	*p*
PCT	3.83 ± 23.35	1.01 ± 3.69	1.39	0.166
WBC	9.15 ± 5.15	10.34 ± 4.25	−2.05	**0.041**
NE	69.41 ± 17.08	76.96 ± 14.36	−3.87	**<0.001**
LY	21.62 ± 13.53	14.24 ± 10.72	4.88	**<0.001**
IL-6	119.64 ± 324.19	90.44 ± 166.72	0.93	0.353
CRP	32.80 ± 50.61	51.91 ± 59.00	−2.81	**0.005**
CSFP	967.63 ± 1700.12	2039.50 ± 2383.07	−4.23	**<0.001**
CSFCL	124.85 ± 7.65	121.24 ± 8.36	3.66	**<0.001**
CSFG	3.54 ± 1.69	3.26 ± 2.02	1.24	0.215
CSF cells	5550.51 ± 24052.14	45855.79 ± 218370.99	−2.15	**0.034**
CSF WBC	1307.82 ± 10331.75	1995.86 ± 11609.70	−0.51	0.612
Fever, *n* (%)			χ^2^ = 4.39	**0.036**
No	55 (43.31)	77 (56.20)		
Yes	72 (56.69)	60 (43.80)		
Headache, *n* (%)			χ^2^ = 0.06	0.802
No	62 (48.82)	69 (50.36)		
Yes	65 (51.18)	68 (49.64)		
Nausea/Vomiting, *n* (%)			χ^2^ = 0.47	0.493
No	73 (57.48)	73 (53.28)		
Yes	54 (42.52)	64 (46.72)		
Altered Consciousness, *n* (%)			χ^2^ = 12.03	**<0.001**
No	53 (41.73)	30 (21.90)		
Yes	74 (58.27)	107 (78.10)		
Meningeal Irritation, *n* (%)			χ^2^ = 5.35	**0.021**
No	88 (69.29)	76 (55.47)		
Yes	39 (30.71)	61 (44.53)		
Cerebral Hemorrhage, *n* (%)			χ^2^ = 52.46	**<0.001**
No	120 (94.49)	76 (55.47)		
Yes	7 (5.51)	61 (44.53)		
Hydrocephalus, *n* (%)			χ^2^ = 26.03	**<0.001**
No	126 (99.21)	109 (79.56)		
Yes	1 (0.79)	28 (20.44)		
Diabetes, *n* (%)			χ^2^ = 2.53	0.111
No	104 (81.89)	101 (73.72)		
Yes	23 (18.11)	36 (26.28)		
Hypertension, *n* (%)			χ^2^ = 9.25	**0.002**
No	71 (55.91)	51 (37.23)		
Yes	56 (44.09)	86 (62.77)		

t, t-test, χ^2^, Chi-square test.

SD, standard deviation. Bold values indicate *p* < 0.05.

### 3.3 Multivariate logistic regression analysis

**Figure 1:**
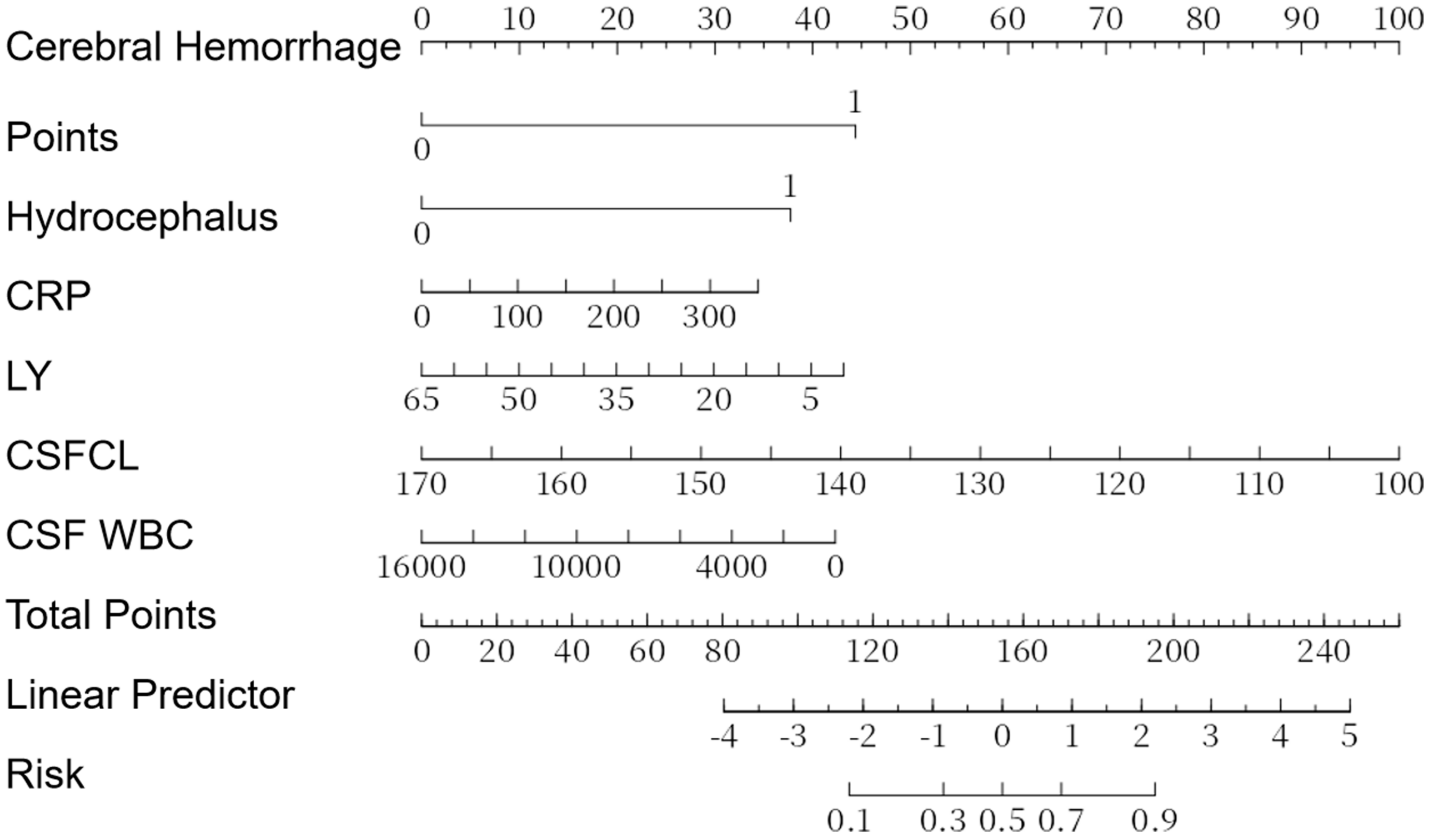
Nomogram of the diagnostic prediction model for purulent meningitis.

The screened variables were used as independent variables, and the presence of Bacterial meningitis was used as the dependent variable to construct the logistic regression model. The steps were as follows: 1. Model training: the data were randomly divided into training and validation sets (7:3 ratio). The training set was used to estimate regression coefficients via maximum likelihood estimation and establish the logistic regression equation. 2. Variable screening: independent variables with a significant impact (*p* < 0.2) were selected using backward stepwise regression. The final model included intracerebral hemorrhage, hydrocephalus, CRP, LY, CSFCL, and cerebrospinal fluid white blood cell count (Table 3). The results of logistic regression analysis were used to construct a nomogram to visualize the risk of bacterial meningitis in patients (Figure 1).

**Table 3:** Results of univariate and multivariate regression analysis.

Variables	Univariate					Multivariate				
	β	S. E	Z	*p*	OR (95%CI)	β	S. E	Z	*p*	OR (95%CI)
Fever										
No					1.00					
Yes	−0.52	0.30	−1.76	0.078	0.59 (0.33 ~ 1.06)					
Headache										
No					1.00					
Yes	−0.13	0.30	−0.44	0.658	0.88 (0.49 ~ 1.57)					
Meningeal Irritation										
No					1.00					
Yes	0.57	0.31	1.84	0.066	1.77 (0.96 ~ 3.26)					
Cerebral Hemorrhage										
No					1.00					1.00
Yes	2.73	0.51	5.40	**<0.001**	15.27 (5.67 ~ 41.09)	2.40	0.55	4.34	**<0.001**	11.01 (3.73 ~ 32.50)
Hydrocephalus										
No					1.00					1.00
Yes	3.03	1.04	2.91	**0.004**	20.63 (2.68 ~ 158.60)	2.04	1.13	1.81	0.071	7.69 (0.84 ~ 70.11)
Diabetes										
No					1.00					
Yes	0.73	0.36	2.05	**0.040**	2.08 (1.03 ~ 4.18)					
Hypertension										
No					1.00					
Yes	0.67	0.30	2.22	**0.026**	1.96 (1.08 ~ 3.54)					
PCT	−0.04	0.06	−0.69	0.491	0.96 (0.84 ~ 1.09)					
IL-6	0.00	0.00	0.26	0.794	1.00 (1.00 ~ 1.00)					
CRP	0.01	0.00	2.72	**0.007**	1.01 (1.01 ~ 1.01)	0.01	0.00	1.40	0.161	1.01 (1.00 ~ 1.01)
WBC	0.07	0.04	1.90	0.058	1.07 (1.00 ~ 1.14)					
NE	0.03	0.01	3.16	**0.002**	1.03 (1.01 ~ 1.06)					
LY	−0.06	0.02	−3.92	**<0.001**	0.94 (0.92 ~ 0.97)	−0.04	0.02	−2.13	**0.034**	0.96 (0.93 ~ 0.99)
CSFP	0.01	0.00	3.21	**0.001**	1.01 (1.01 ~ 1.01)					
CSFCL	−0.06	0.02	−2.87	**0.004**	0.94 (0.91 ~ 0.98)	−0.08	0.03	−3.02	**0.003**	0.93 (0.88 ~ 0.97)
CSFG	0.00	0.08	0.02	0.987	1.00 (0.86 ~ 1.17)					
CSF cells	0.00	0.00	1.77	0.077	1.00 (1.00 ~ 1.00)					
CSF WBC	0.00	0.00	1.54	0.122	1.00 (1.00 ~ 1.00)	−0.00	0.00	−1.47	0.141	1.00 (1.00 ~ 1.00)

Bold values indicate *p* < 0.05.

### 3.4 Model evaluation

**Figure 2:**
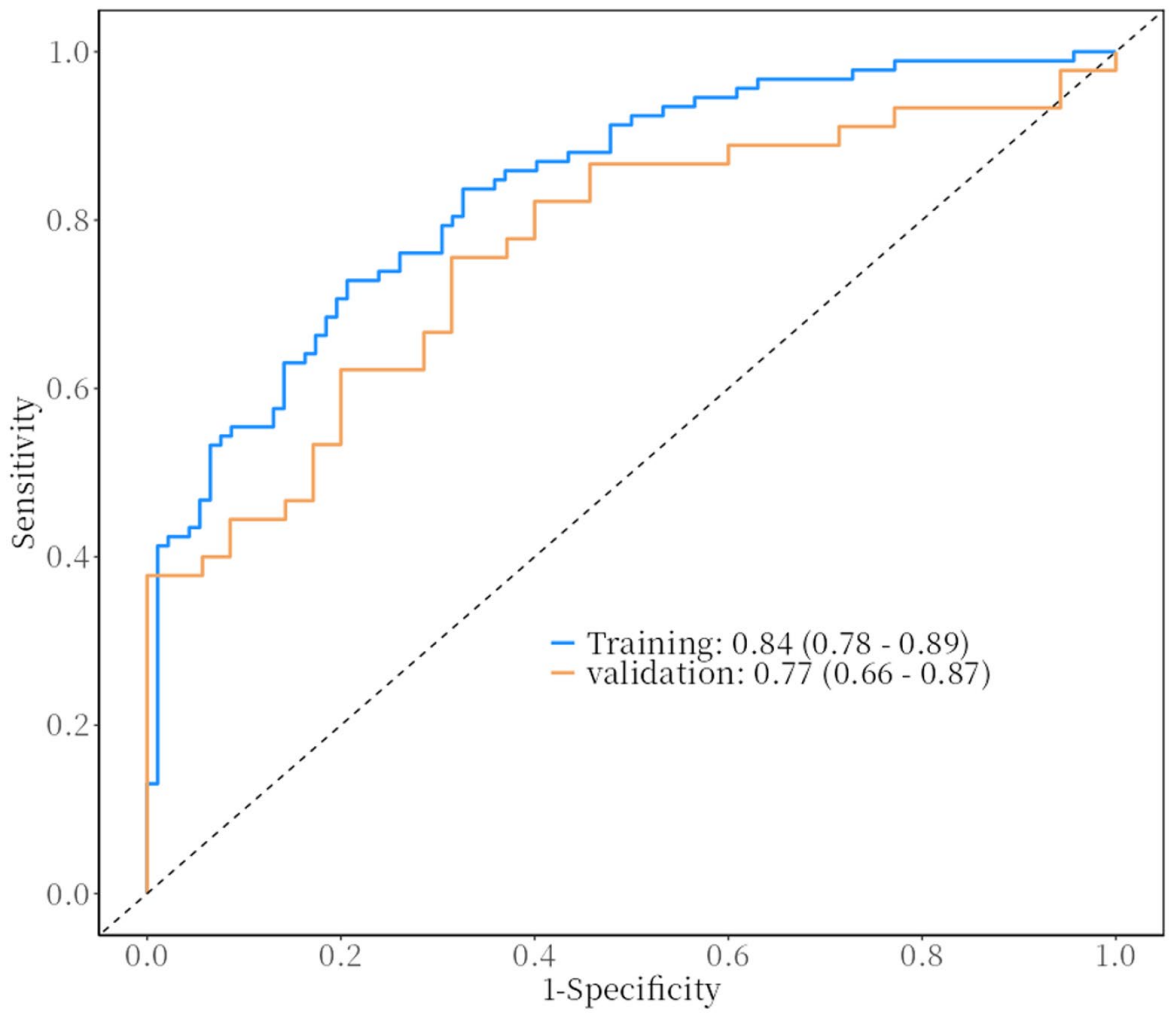
The AUC for the training set was 0.84 (95% CI: 0.78–0.89). The AUC for the validation set was 0.77 (95% CI: 0.66–0.87).

**Figure 3:**
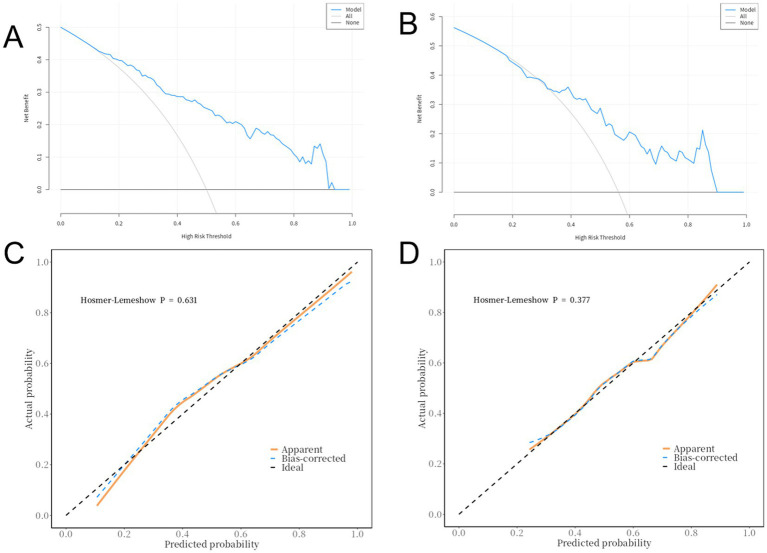
DCA of the training set **(A)** and validation set **(B)**, H-L test of the training set **(C)** and validation set **(D)**.

The ROC curve for the training set was close to the top-left corner, with an AUC of 0.84 (95% CI: 0.78–0.89). The validation set also performed well, with an AUC of 0.77 (95% CI: 0.66–0.87), indicating good discriminative ability (Figure 2). Decision curve analysis (DCA) showed that the model had good net benefits across different risk thresholds for both the training set (Figure 3A) and validation set (Figure 3B). The calibration curve for the training set had a mean absolute error of 0.031 (Figure 3C), and the validation set had a mean absolute error of 0.017 (Figure 3D). The logistic regression model was evaluated using the Hosmer-Lemeshow (H-L) test to assess goodness-of-fit for the training and validation sets. Training Set: *p* = 0.6314. The *p*-value > 0.05 indicated good fit, with no significant difference between predicted and observed values. Validation Set: *p* = 0.3768. Similarly, the p-value > 0.05 indicated good fit, with no significant difference between predicted and observed values indicating close alignment between predicted and actual results. The model’s accuracy was 0.76 (95% CI: 0.69–0.82) for the training set and 0.69 (95% CI: 0.57–0.79) for the validation set. Sensitivity was 0.79 (95% CI: 0.71–0.88) and specificity was 0.73 (95% CI: 0.64–0.82) for the training set, while sensitivity was 0.71 (95% CI: 0.56–0.86) and specificity was 0.67 (95% CI: 0.53–0.80) for the validation set. Positive predictive value (PPV) was 0.74 (95% CI: 0.66–0.83) and negative predictive value (NPV) was 0.78 (95% CI: 0.69–0.87) for the training set, while PPV was 0.62 (95% CI: 0.47–0.78) and NPV was 0.75 (95% CI: 0.62–0.88) for the validation set (Table 4).

**Table 4:** Confusion matrix of the training and validation sets.

Data	AUC (95%CI)	Accuracy (95%CI)	Sensitivity (95%CI)	Specificity (95%CI)	PPV (95%CI)	NPV (95%CI)	cut off
Training set	0.84 (0.78–0.89)	0.76 (0.69–0.82)	0.79 (0.71–0.88)	0.73 (0.64–0.82)	0.74 (0.66–0.83)	0.78 (0.69–0.87)	0.453
Validation set	0.77 (0.66–0.87)	0.69 (0.57–0.79)	0.71 (0.56–0.86)	0.67 (0.53–0.80)	0.62 (0.47–0.78)	0.75 (0.62–0.88)	0.453

### 3.5 How to use the bacterial-meningitis nomogram

Bedside application of the nomogram takes < 2 min: score each predictor by dropping a perpendicular from the patient’s value to the Points axis, sum the six scores, then read the corresponding probability on the bottom scale. A risk ≥ 15% indicates immediate empirical antibiotics ± repeat CSF or short-interval imaging; a risk < 15% prompts senior review with rescue therapy if clinical deterioration occurs. For example, a 55-year-old man with fever, neck stiffness and confusion, CSF WBC = 2000 cells μL^−1^ (35 points), CSF chloride = 120 mmol L^−1^ (20 points), lymphocyte % = 10% (15 points), CRP = 50 mg L^−1^ (25 points), intracerebral haemorrhage = Yes (100 points), hydrocephalus = No (0 points), gives a total of 195 points corresponding to a probability ≈ 68%; since risk ≥ 15%, maintain empirical antibiotics and schedule follow-up CT within 12 h.

### 3.6 Etiological results

Among the 137 Bacterial meningitis cases, cerebrospinal fluid bacterial cultures were positive in 111 cases. Gram-negative bacteria accounted for 17 cases (15.3%), and Gram-positive bacteria accounted for 94 cases (84.7%). Drug Resistance Analysis:

Gram-Positive Bacteria: 46 strains were *β*-lactamase-positive, resistant to penicillin, amino-, carboxy-, and ureido-penicillins.39 strains were methicillin-resistant Staphylococcus (MRS), resistant to penicillins, β-lactam/β-lactamase inhibitor combinations, cephalosporins, and carbapenems. - Gram-Negative Bacteria: 9 strains included 1 carbapenem-resistant Enterobacteriaceae (CRE), 1 colistin-resistant *Acinetobacter baumannii* (CR-Aba), 1 New-Delhi metallo-β-lactamase-producing isolate (NDM), 4 high-level aminoglycoside-resistant enterococci (HLAR), and 1 vancomycin-resistant enterococcus (VRE) (Table 5).

**Table 5:** Pathogen distribution of 111 cases with positive cerebrospinal fluid bacterial culture.

Pathogen	Number (*n* = 111)	Proportion (%)
Gramnegative bacteria	17	15.3
*Klebsiella pneumoniae*	1	0.9
*Escherichia coli*	1	0.9
*Corynebacterium striatum*	2	1.8
Arthrobacter radiobacter	1	0.9
Nonfermenting Corynebacterium	1	0.9
Confusis Corynebacterium	1	0.9
Urealyticum Corynebacterium	1	0.9
*Corynebacterium bovis*	1	0.9
*Acinetobacter baumannii*	1	0.9
*Enterobacter asburiae*	1	0.9
*Citrobacter freundii*	1	0.9
*Aeromonas hydrophila*	1	0.9
*Pseudomonas fluorescens*	1	0.9
*Pseudomonas putida*	1	0.9
Others	2	1.8
Grampositive bacteria	94	84.7
*Staphylococcus aureus*	9	8.1
*Staphylococcus hominis*	25	22.5
*Staphylococcus epidermidis*	20	18.0
Staphylococcus hemolyticus	9	8.1
*Staphylococcus capitis*	9	8.1
*Staphylococcus cohnii*	1	0.9
*Enterococcus faecium*	7	6.3
*Enterococcus faecalis*	3	2.7
*Streptococcus pneumoniae*	2	1.8
*Neisseria meningitidis*	1	0.9
*Streptococcus mitis*	1	0.9
*Micrococcus luteus*	3	2.7
*Listeria monocytogenes*	1	0.9
*Microbacterium aurum*	1	0.9
Others	2	1.8

## 4 Discussion

This research developed a predictive model for diagnosing bacterial meningitis by utilizing clinical and laboratory information through univariate and logistic regression techniques. The resulting model exhibited strong diagnostic capabilities, achieving notable accuracy, sensitivity, specificity, and AUC metrics. Research shows confirmed that CSF CRP is an excellent diagnostic marker for bacterial meningitis, and that its implementation in clinical practice is simple and available at low cost. This makes it a valuable tool for diagnosing bacterial meningitis by enabling timely and accurate treatment, which is essential for a good prognosis (21). Research shows CRP, IL-6, showed the largest mean differences between bacterial meningitis and viral meningitis and IL-6 pct between bacterial meningitis and no CNS infection/inflammation (22). Diagnosing NBM is particularly difficult because diagnosis relies on CSF culture results, which have high false-negative rates relates to the high utilization rate of antimicrobial drugs (23, 24). Patients have often been prescribed antibiotics at the outpatient level, which increases the likelihood that CSF cultures produce false-negative results (25). Some researchers have found that antibiotic treatment prior to admission reduces CSF culture positivity rates by nearly 30% (26). Although we supplemented culture with 16 S rRNA PCR and metagenomic next-generation sequencing (mNGS), false-negative bacterial cases may have been misclassified as NBM, artificially lowering the apparent specificity of our model. Future multicentre prospective cohorts should standardize the timing of lumbar puncture and record outpatient antibiotic exposure so that culture-negative, PCR-positive cases can be analysed separately. The model was derived from a single tertiary-care centre. Age distribution, referral patterns and local anti-microbial stewardship policies may limit external validity. For example, hydrocephalus – a key predictor in our nomogram – is probably over-represented in a referral neurosurgical population. The study was conducted under intermediate–high antimicrobial-pressure conditions. Re-training the model in settings with low pre-test probability (e.g., primary-care hospitals) or emerging drug-resistant epidemiology will be necessary. Transfer-learning techniques can accelerate this process by retaining the core logistic structure while updating only the coefficient layer.

Although decision-curve analysis confirmed net clinical benefit across threshold probabilities of 10–60%, the lower limit of the 95% CI for validation-set sensitivity (0.56–0.86) implies that up to 29% of true bacterial meningitis cases could still be missed. We therefore recommend that the nomogram be used as a triage—rather than a rule-out—tool. Patients scoring above the 15% risk threshold should receive empirical antibiotics while awaiting culture or mNGS results; those below the threshold require senior clinician review and, if feasible, repeat lumbar puncture within 6–12 h. However, the study has limitations, including single-center data collection, potential selection bias, and the exclusion of imaging data. Future research should validate the model in multicenter studies and incorporate additional diagnostic information to enhance clinical utility and generalizability.

Our nomogram attained AUC of 0.84 in the training and 0.77 in the validation cohort, surpassing the conventional bacterial meningitis score (BMS; AUC 0.72) derived from a Korean adult series of 614 acute meningitis cases (16). The BMS incorporates only cerebrospinal fluid (CSF) glucose, protein, neutrophil fraction and serum C-reactive protein (CRP); by additionally including intracerebral haemorrhage and CSF chloride—both obtainable within 1 h of lumbar puncture—we achieved an absolute 8% gain in AUC and a number-needed-to-diagnose of 4 (95% CI 3–6). A Dutch paediatric investigation that combined CSF CRP with interleukin-6 achieved an AUC of 0.83 (22); however, IL-6 is rarely assayed in medium-volume Chinese hospitals. Our model replaces IL-6 with peripheral-blood lymphocyte percentage and CSF chloride, parameters routinely supplied by standard haematology and electrolyte analysers at no extra cost. Wang et al. recently identified neuro-imaging evidence of intracranial haemorrhage as an independent predictor of bacterial meningitis in a multicentre Chinese study (odds ratio 5.4, 95% CI 2.3–12.7) (20); we confirm and quantify this observation, assigning intracerebral haemorrhage the highest weight (100 points) in our nomogram, corresponding to an adjusted odds ratio of 11.01 after accounting for hydrocephalus and inflammatory markers. Concordance between imaging and laboratory predictors in two independent Chinese cohorts reinforces the generalisability of intracranial haemorrhage as a discriminatory variable. In a UK single-centre adult study, Olie et al. validated CSF CRP ≥ 1 mg L^−1^ as a rule-in criterion (specificity 93%, sensitivity 71%) (21); application of this threshold to our validation set yielded similar specificity (90%) but reduced sensitivity (58%), partly because 31% of our patients had received outpatient antibiotics, thereby lowering CSF CRP concentrations. Integration of CSF CRP with five additional variables in our nomogram restored sensitivity to 71% without compromising specificity (67%), demonstrating that a multiparameter model is more robust than any single biomarker when prior antibiotic exposure attenuates pre-test probability. Collectively, these comparisons indicate that the present model achieves higher discriminative accuracy than previous scores while employing inexpensive, rapidly available parameters that remain stable across different age groups and healthcare settings; nevertheless, prospective multicentre validation is warranted before clinical deployment outside tertiary-care facilities.

In summary, we developed an interpretable, culture-independent diagnostic model that outperformed the traditional BMS score (AUC 0.84 vs. 0.72) by incorporating hydrocephalus and CSF chloride. The nomogram allows 10-min bedside risk stratification with routine laboratory tests, supporting early antibiotic decisions when culture is pending. Multicentre, prospective validation and integration of imaging and host-transcriptomic biomarkers will be the next steps toward universal clinical deployment.

## 5 Conclusion

This research created a predictive model for diagnosing bacterial meningitis utilizing both clinical and laboratory information, such as intracerebral hemorrhage, hydrocephalus, CRP, LY, CSFCL, and the white blood cell count in cerebrospinal fluid, which showed strong diagnostic accuracy. The model offers a probabilistic evaluation of bacterial meningitis risk and has proven effective in prediction and clinical use. Future investigations will aim at validating the model across multiple centers and enhancing its clinical relevance and generalizability. Our model achieved an AUC of 0.84, surpassing the BMS score of 0.72 by incorporating innovative predictors like hydrocephalus and CSFCL. The nomogram facilitates quick risk assessment within 10 min using standard tests, supporting antibiotic treatment decisions while awaiting culture results.

## Data Availability

The original contributions presented in the study are included in the article/supplementary material, further inquiries can be directed to the corresponding author.
